# Free-Surface Velocity Measurement Using Direct Sensor Orientation-Based STIV

**DOI:** 10.3390/mi13081167

**Published:** 2022-07-23

**Authors:** Zhen Zhang, Lijun Zhao, Boyuan Liu, Tiansheng Jiang, Ze Cheng

**Affiliations:** College of Computer and Information Engineering, Hohai University, Nanjing 211100, China; 211607010145@hhu.edu.cn (L.Z.); 1806020329@hhu.edu.cn (B.L.); 211607010039@hhu.edu.cn (T.J.); 211607010013@hhu.edu.cn (Z.C.)

**Keywords:** PIV, STIV, velocity measurement, GCPs-free, direct sensor orientation

## Abstract

Particle image velocimetry (PIV) is a quantitative flow visualization technique, which greatly improves the ability to characterize various complex flows in laboratory and field environments. However, the deployment of reference objects or ground control points (GCPs) for velocity calibration is still a challenge for in situ free-surface velocity measurements. By combining space-time image velocimetry (STIV) with direct sensor orientation (DSO) photogrammetry, a laser distance meter (LDM)-supported photogrammetric device is designed, to realize the GCPs-free surface velocity measurement under an oblique shooting angle. The velocity calibration with DSO is based on the collinear equation, while the lens distortion, oblique shooting angle, water level variation, and water surface slope are introduced to build an imaging measurement model with explicit physical meaning for parameters. To accurately obtain the in situ position and orientations of the camera utilizing the LDM and its embedded tilt sensor, the camera’s intrinsic parameters and relative position within the LDM are previously calibrated with a planar chessboard. A flume experiment is designed to evaluate the uncertainty of optical flow estimation and velocity calibration. Results show that the proposed DSO-STIV has good transferability and operability for in situ measurements. It is superior to propeller current meters and surface velocity radars in characterizing shallow free-surface flows; this is attributed to its non-intrusive, whole-field, and high-resolution features. In addition, the combined uncertainty of free-surface velocity measurement is analyzed, which provides an alternative solution for error assessment when comparing measurement failures.

## 1. Introduction

Particle image velocimetry (PIV) is a quantitative flow visualization technique [1]. It seeds particles with good light scattering to trace their flow and estimates their motion as displacement or velocity vectors between two successive frames via image processing to present the flow field. Compared with traditional velocity measurement methods, it greatly improves the ability to characterize various complex flows in laboratory and field environments; this is attributed to its non-intrusive and whole-field measurement features [2]. Velocity calibration, which transforms the motion vectors in the image coordinate system to the velocity vectors in the world coordinate system, is one of the key issues in PIV [3].

In laboratory flume and river model experiments, cameras generally shoot perpendicular to the test plane. Under these conditions, the scaling relation for orthographic projection could be simply calculated with known-size reference objects set on the test plane [4,5]. A more rigorous approach is to build the invertible coordinate transformation relation between the image plane and the object plane by solving the homography matrix. The direct linear transformation (DLT) is a commonly used method in close-range photogrammetry, which needs at least four non-aligned ground control points (GCPs) on the free surface [6]. The planar homography model is simple and GCPs must be strictly coplanar with the test plane. Otherwise, their projections on the image cannot represent the true elevation of the free surface to be measured. However, these conditions are often difficult to control in practice, resulting in calibration error [7].

In contrast, the conditions of large-scale river surfaces are more complicated. Firstly, in order to survey the river cross-section as completely as possible, the camera needs to be installed on a riverbank and shoot with a small pitch angle to obtain a large field of view, ranging from hundreds to thousands of square meters. This can lead to serious image perspective distortion and the loss of spatial resolution in the far-field image. Image ortho-rectification can correct the perspective distortion [8,9,10,11]; however, it not only increases the consumption of computation and memory, but also brings in errors induced by gray level interpolation [12]. Secondly, the water level of natural rivers varies quickly and significantly, especially in mountain streams where the variation can be several meters in minutes. This can change the projective relation of the free surface, thereby affecting the scale and position of the measuring area [13]. Moreover, it is quite difficult to deploy GCPs on the river surface [14]. Some studies have tried to establish the projective relation with a floating calibration board [15] or laser points tracing the water surface [16]. This can meet the measurement requirement of flow fields ranging from tens to hundreds of square meters under a large shooting angle. However, these reference objects often have poor visibility under complex illumination conditions such as shadows and glares on the river surface, which makes it difficult to automatically detect and accurately locate objects within the image. The detection error will be significantly amplified when applied to a large-scale area [17]. Currently, the commonly used velocity calibration scheme is based on variable height homography (VHH) [7,18,19,20]. It improves the measuring precision of bank-based large-scale PIV (LSPIV) systems with small shooting angles by considering the distance from GCPs to the free surface, as well as modeling the elevation of the camera with the water level and the river slope. A limitation of VHH is that at least six non-coplanar control points should be distributed evenly on both sides of the river and surveyed by a total station or differential global positioning system (DGPS). It is a labor-consuming and high-risk task, especially in flood emergency monitoring. In addition, it is found that solving VHH with DLT is sensitive to the number, distribution, and accuracy of GCPs, as well as lens distortion. The solving process trends to be non-convergence under unfavorable conditions and fails to build the coordinate transformation relation [21]. Therefore, the velocity calibration problem has become one of the main bottlenecks of LSPIV.

The two-step method [22] for camera calibration in the computer vision society provides a solution for reducing the dependence of PIV on GCPs. It takes into account the nonlinear distortion of the lens and decomposes the homography matrix into intrinsic and extrinsic camera matrices. The photogrammetric task is divided into two steps: (1) indoor calibration of intrinsic parameters (focal length and principal point) and distorted aberration (radial and tangential distortion); and (2) in situ calibration of extrinsic parameters (translation and rotation). This not only reduces the number of GCPs required by the in situ calibration to four [23], but also improves the flexibility of system deployment. For example, a rotational calibration technique based on precise pan-tilt heads is devised to ensure accurate camera geometry in the wide river setting [24,25]. It can utilize near-field GCPs with better visibility to calibrate the initial value of the extrinsic parameters. The camera can then be rotated to the measuring position and can compensate for its orientations (azimuth and pitch angles) via the readings of two dials on the pan-tilt heads. Since the above calibration process requires manual adjustment and reading, automatic measurement has not been supported so far. When the camera orientation changes due to external disturbance (such as a storm) the present calibration results will no longer be applicable. Re-surveying GCPs and re-calibrating may cause inconsistencies in the world reference system, which creates problems for data use. Obviously, the development of GCPs-free imaging velocimetry has important significance for online river flow monitoring.

With the advances in sensor and information fusion technology, the positioning and orientation system (POS) is explored for extrinsic parameter measurement and coordinate transformation in both land-based [26] and aerial photogrammetry fields [27]. This technique is known as direct sensor orientation (DSO) or direct georeferencing (DG). At present, it can realize the GCPs-free measurement with decimeter-level precision within several square kilometers. The measurement quality of these systems is highly dependent on the precision of POS. This limitation is a major drawback due to the elevated cost associated with high-end POS units, particularly the inertial system. Generally, it is required that the roll and pitch angle errors of inertial systems (INS) should not be greater than 0.01°, the azimuth angle error should not be greater than 0.02°, and the recording frequency should be higher than 50 Hz. The positioning accuracy of GPS should reach the centimeter level, and the minimum sampling interval is generally less than one second. In addition, the potential accuracy of the DSO also depends on the architecture and quality of the GPS/INS integration process, as well as the validity of the system calibration. However, the calibration of multiple sensors, as well as the system mounting parameters (i.e., six elements of exterior orientation), is a delicate and complicated task. For the aerial photogrammetric systems, a special ground control field generally needs to be deployed for regular in-flight calibration. Fortunately, for PIV systems with fixed cameras, the position can be measured after installation and the orientation can be synchronously measured by a tilt sensor when capturing images. The DSO-based PIV has the potential to utilize low-cost sensors and improve the efficiency and safety of in situ measurements.

In this paper, a method which combines DSO and space-time image velocimetry (STIV) is proposed to realize free-surface velocity measurement without GCPs and image ortho-rectification. Both the safety and efficiency of field operations can be effectively improved using this low-cost measurement method. Section 2 firstly introduces the principle of an FFT-STIV based on the fast Fourier transform (FFT), then mainly focuses on the velocity calibration using DSO. Section 3 introduces the design of a laser distance meter (LDM)-supported measurement device and mainly focuses on its calibration. Section 4 presents a flume experiment to validate the method when applied to a small-scale free surface and evaluates its uncertainty. Section 5 concludes the paper and proposes research needs for future work.

## 2. Principle of DSO-Based FFT-STIV

### 2.1. Motion Estimation with FFT-STIV

**Figure 1:**
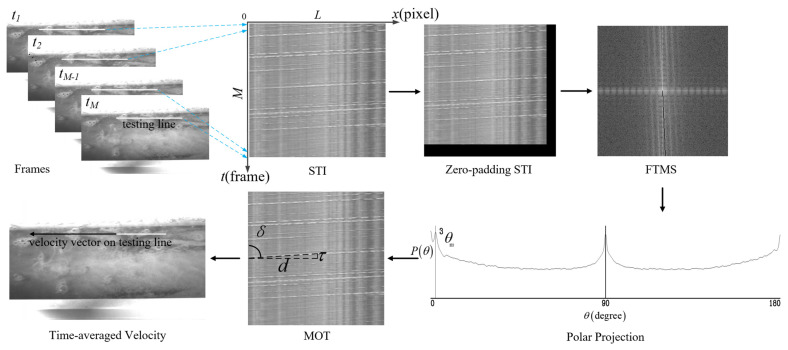
Outline of FFT-STIV.

STIV is a motion estimation method for time-averaged optical flow [28]. It assumes that the tracers that follow the surface flow satisfy the continuity of motion within a short time. It makes their position in the two-dimensional (2-D) space-time image (STI) present as a significant directional texture, with a main orientation that is a function of the one-dimensional (1-D) time-averaged velocity on a testing line. Compared to LSPIV, its spatial resolution can achieve single-pixel level, and its computational efficiency is above 10 times than that of the correlation-based method [29]. This makes it particularly suitable for real-time velocity-field observation under a small shooting angle. In this study, the central perspective images (rather than the ortho-rectified ones) are directly used for motion estimation to avoid additional errors. Figure 1 illustrates the outline of the FFT-STIV.

Frames of *M* are captured from a camera or a video with time intervals of Δt seconds, in which a testing line with the length of L pixels is set along the flow direction. The testing line can be regarded as the interrogation area (IA) with the width of single pixel. An STI the size of L × M (pixels) can be generated in x-t coordinates. It has a directional texture resembling bright and dark bands formed by the motion of optical flow on the free surface. Assuming that the motion of optical flow is equivalent to the motion of flow tracers, the main orientation of texture (MOT), which is defined as δ, can reflect the magnitude and direction of the time-averaged velocity V (m/s) during time interval of M × Δt(s) on the testing line:

(1) $$V=\frac{D}{T}=\frac{d\;\cdot \;S}{\tau \;\cdot \;\Delta t}=\tan\;\delta \;\cdot \;\frac{S}{\Delta t}=v\;\cdot \;S$$

where D (m)represents the distance that tracers traveled in physical coordinates within T (s), d (pixel)represents the distance that tracers traveled in image coordinates within τ (frame), v (pixel/s) represents the optical flow velocity and its sign denotes the motion direction, s (m/pixel)represents the scaling factor (i.e., the spatial resolution) on the testing line, and δ (°) represents the main orientation of texture.

To overcome the deficiencies under deteriorated light (shadows and glares) and natural seeding conditions (sparse and uneven spatiotemporal distribution), the study attempts to address the issue of MOT detection in the frequency domain according to the auto-registration property of the Fourier transform magnitude spectra F(u,v). This auto-registration depends on the orientation and variation frequency of the image texture, rather than their spatial locations. It makes the FFT-STIV robust to local noises and randomly occurring tracers. The 2-D FFT of STI is noted below:

(2) $$F(u\;,\;v)=\mathrm{FFT}2(\mathrm{STI}(x\;,\;y)=\frac{1}{N^{2}}\overset{N-1}{\underset{x=0}{\sum }}\overset{N-1}{\underset{y=0}{\sum }}{\mathrm{STI}(x\;,\;y)e}^{-j2\pi (\frac{xu+yv}{N})}$$

where FFT2 represents the two-dimensional fast Fourier transform, STI(*x*, *y*) represents the space-time image, and N represents the size of the space-time image.

To avoid “spectrum compression” caused by unequal width and height, the STI is enlarged to the size of N × N pixels via zero-padding. The texture patterns of STI are re-distributed in lines passing through the center of its centrosymmetric FTMS, and the main orientation of spectra (MOS) θ is orthogonal to the MOT:

(3) $$\delta =\theta \;-{\;90}^{{}^{\circ}}$$

where δ(°) represents the main orientation of texture.

To detect the MOS, the F(u,v) is projected to the polar coordinate system where the polar radius r=N/2 pixels and the polar angle ranges from 0° to 180°:

(4) $$P(\theta )=\overset{R}{\underset{r=0}{\sum }}|F(r\;,\;\theta )|\;/\;N$$

The $\theta_{m}$ is determined further by searching and Gaussian fitting the peak value of P(θ):

(5) $$\theta_{m}=\arg\;\max[P(\theta )]$$

The low frequency background noises are mainly distributed within ±5° around 0° and 90° in the polar projection. Alternative high-pass filtering, or the edge detection procedure, is recommended for background suppression [30] when peak detection of P(θ) is interfered with by a low signal-to-noise ratio.

### 2.2. Velocity Calibration with DSO

**Figure 2:**
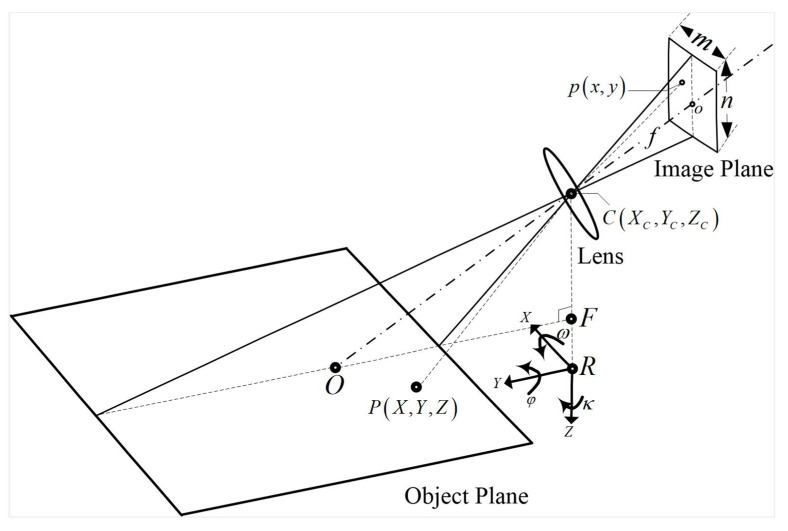
Model of central projection imaging under an oblique view.

Velocity calibration aims to convert the optical flow velocity into the physical velocity and determine the position (starting distance) of the testing line. The essential issue is how to establish the coordinate transformation relation between the object plane and the image plane. The central perspective projection model in photogrammetry provides a theoretical basis for solving such problems. In Figure 2, the object point P(X,Y,Z), projection center ${C(X}_{C}{,Y}_{C}{,Z}_{C})$, and image point P(X,Y) are assumed collinear; o and O are the projection points of ${C(X}_{C}{,Y}_{C}{,Z}_{C})$ on the image plane and the object plane, respectively. For a perfect pinhole imaging system, the principal point o is at the center of the image, with image plane coordinates (in mm) of:

(6) $$\{\begin{array}{c} x_{o}=s\;\cdot \;m\;/\;2\\ y_{o}=s\;\cdot \;n\;/\;2 \end{array}$$

where s (mm) is the pixel size of the image sensor and m × n (pixels) is the image size. The image plane coordinates (x,y) are expressed by their image coordinates (i,j) (pixel) as:

(7) $$\{\begin{array}{c} x_{o}=s\;\cdot \;i\\ y_{o}=s\;\cdot \;j \end{array}$$

Considering the object distance (OC) is much larger than the image distance (oc), the focal length f can be assumed equal to the image distance. The linear geometric relation between image points and object points is established by similar triangles. If the image plane and the object plane coordinate systems are taken as the reference, respectively, the collinear equation can be expressed in the following direct and indirect forms:

(8) $$\{\begin{array}{c} {x=x}_{o}-f\frac{a_{1}\left(X\;-{\;X}_{c}\right){+b}_{1}\left(Y\;-{\;Y}_{c}\right){+c}_{1}\left(Z\;-{\;Z}_{c}\right)}{a_{3}\left(X\;-{\;X}_{c}\right){+b}_{3}\left(Y\;-{\;Y}_{c}\right){+c}_{3}\left(Z\;-{\;Z}_{c}\right)}\\ {y=y}_{o}-\;f\frac{a_{2}\left(X\;-{\;X}_{c}\right){+b}_{2}\left(Y-{\;Y}_{c}\right){+c}_{2}\left(Z\;-{\;Z}_{c}\right)}{a_{3}\left(X\;-{\;X}_{c}\right){+b}_{3}\left(Y\;-{\;Y}_{c}\right){+c}_{3}\left(Z\;-{\;Z}_{c}\right)} \end{array}$$

(9) $$\{\begin{array}{c} X=\frac{a_{1}(x\;-{\;x}_{o}{)+a}_{2}(y\;-{\;y}_{o}{)+a}_{3}(-\;f)}{c_{1}(x\;-{\;x}_{o}{)+c}_{2}(y\;-{\;y}_{o}{)+c}_{3}(-\;f)}(Z\;-{\;Z}_{c}{)+X}_{c}\\ Y=\frac{b_{1}(x\;-{\;x}_{o}{)+b}_{2}(y\;-{\;y}_{o}{)+b}_{3}(-\;f)}{c_{1}(x\;-{\;x}_{o}{)+c}_{2}(y\;-{\;y}_{o}{)+c}_{3}(-\;f)}(Z\;-Z_{c}{)+Y}_{c} \end{array}$$

where f represents the focal length and *Z* represents the elevation of the object plane.

The above two equations describe the reciprocal transformation relation of two plane coordinates. The direct form projects object point (X,Y,Z) to image point (x,y). The indirect form projects image point (x,y) to object point (X,Y), but the Z point coordinate is required. The rotation matrix formed by nine coefficients in the equations can be expressed by the camera’s orientation angles relative to the object’s 3D coordinate system:

(10) $$\begin{array}{c} R^{T}=\left[\begin{array}{ccc} a_{1} & b_{1} & c_{1}\\ a_{2} & b_{2} & c_{2}\\ a_{3} & b_{3} & c_{3} \end{array}\right]=\\ \left[\begin{array}{ccc} \cos\kappa \cdot \cos\varphi -\sin\kappa \cdot \sin\omega \cdot \sin\varphi & \cos\omega \cdot \sin\varphi & \sin\kappa \cdot \cos\varphi +\cos\kappa \cdot \sin\omega \cdot \sin\varphi \\ -\cos\kappa \cdot \sin\varphi -\sin\kappa \cdot \sin\omega \cdot \cos\varphi & \cos\omega \cdot \cos\varphi & -\sin\kappa \cdot \sin\varphi +\cos\kappa \cdot \sin\omega \cdot \cos\varphi \\ -\sin\kappa \cdot \cos\omega & -\sin\omega & \cos\kappa \cdot \cos\omega \end{array}\right] \end{array}$$

where the pitch (ω), roll (φ), and azimuth (κ) angles are defined as the angles used in order to rotate a (X,Y,Z) geodetic coordinate system and align it with the image coordinate system. Pitch is the rotation around the *X* axis. Roll is the rotation around the *Y* axis. Azimuth is the rotation around the *Z* axis.

There are six unknowns ${(X}_{C}{,Y}_{C}{,Z}_{C},\varphi ,\omega ,\kappa )$ to be solved, which are known as the extrinsic parameters of the camera. Existing methods usually utilize at least three non-collinear GCPs to solve the unknowns, and use (8) to transform coordinates and generate ortho-rectified images for subsequent motion estimation. In this study, the model is reasonably simplified considering the fixed camera position. The object coordinate system is established by taking the reference point R on a fixed ground or sea level as the origin. Point F is the pedal point of point C on the measuring plane. The camera’s optical axis is adjusted parallel to the cross-section; thus, it can be assumed that ($X_{C}{,Y}_{C})=(0,0)$ and κ=0. The remaining three extrinsic parameters ${(Z}_{C},\varphi ,\omega )$ are obtained by distance and tilt sensors to realize the DSO-based photogrammetry.

To avoid additional errors and computations induced by image ortho-rectification, the velocity calibration is carried out based on (9) instead of (7). For STIV, the velocity on the testing line is denoted as:

(11) $$V_{i\;,j}{=X}_{{\;i+v}_{x}\;,\;j}-{\;X}_{i\;,j}$$

where $X_{{\;i+v}_{x}\;,\;j}$ and ${\;X}_{i\;,j}$ represent the coordinates of the image point in the object plane.

The location (i.e., starting distance of the cross-section) of the testing line is denoted as:

(12) $$D_{i,j}{=|Y}_{i,j}{|+D}_{C}$$

where $D_{C}$ represents the *Y*-axis projection distance of the point F relative to the reference point. Note that the scaling factor *s* in (1) is not directly solved by the above velocity calibration process. On the contrary, according to its definition, it can be derived using the physical distances between two adjacent pixels.

## 3. Calibration of Measuring Device

### 3.1. Measuring Device

**Figure 3:**
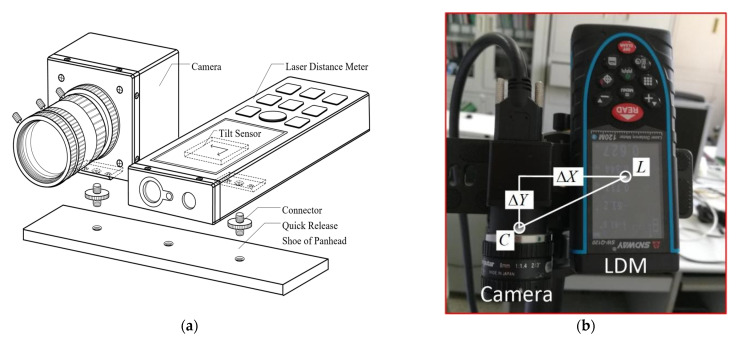
LDM-supported measuring device showing (**a**) structure diagram and (**b**) picture of real device.

An LDM-supported measuring device (Figure 3) was designed for the DSO-based FFT-STIV. A USB 3.0 industrial camera (HIKVISION MV-CA013-20UM, made by Hikvision, in Hangzhou, China) was used for image capture. It had a monochrome CMOS sensor with an image size of 1280 × 1024 pixels and a pixel size of 4.8 μm. The frame rate at full resolution was up to 170 fps. In this study, a C-mount lens with a focal length of 8 mm was installed on the camera. The LDM (SNDWAY SW-Q120, made by SNDWAY, in Dongguan, China) was equipped with an embedded dual-axis (pitch and roll) tilt sensor. The measuring ranges of pitch and roll angles were both ±90° with precision up to 0.1°. The measuring range and precision of distance can reach 120 m and 3 mm, respectively. The camera and LDM were rigidly mounted on a platform (a quick release shoe of panhead) with parallel optical axes. Their geometric relation was assumed to be invariant. The major error sources of the measuring device included the nonlinear distortion aberration of the lens, as well as the eccentric distances and eccentric angles between the camera and the LDM. To reduce their effect, the corresponding calibration methods are discussed below.

### 3.2. Calibration of Distortion Aberration

**Figure 4:**
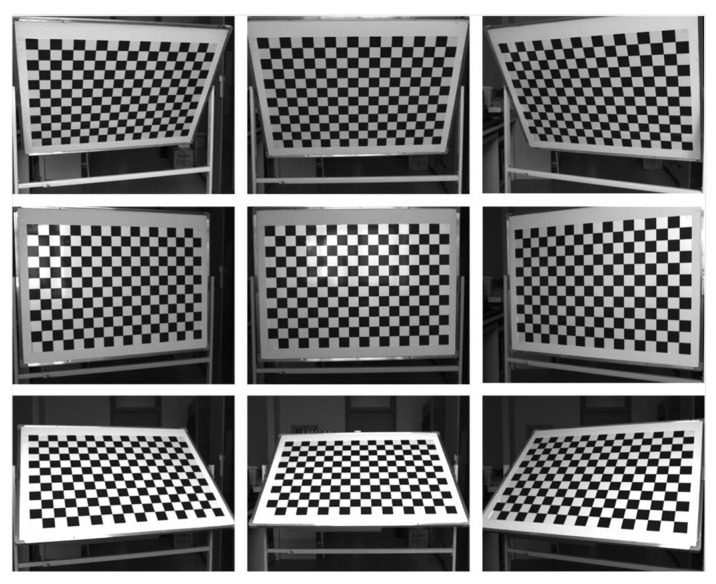
Chessboard images for camera calibration.

Due to the complexity of lens design and manufacture, the real imaging system cannot strictly satisfy the central perspective projection model. This lens distortion deviates the actual image position from that given by the central perspective projection model, especially in the use of wide-angle lenses. In high-precision photogrammetry, a nonlinear imaging model considering distortion aberration should be applied for image correction. Furthermore, the focal length should use the calibrated value rather than the nominal one. In this study, both intrinsic parameters and distortion aberration are calibrated in the laboratory with the planar chessboard method provided by the camera calibration toolbox from OpenCV. A chessboard with 18 × 12 square grids was designed and the side lengths of each grid were 60 mm. A total of nine images taken under different shooting angles were captured for calibration (Figure 4). The intrinsic parameter matrix K and the distortion parameter matrix D were calculated as follows:

(13) $$K=\left[\begin{array}{ccc} f_{x} & 0 & c_{x}\\ 0 & f_{y} & c_{y}\\ 0 & 0 & 1 \end{array}\right]=\left[\begin{array}{ccc} 1740.2670 & 0 & 637.8651\\ 0 & 1743.1978 & 526.1480\\ 0 & 0 & 1 \end{array}\right]$$

(14) $$D=\left[\begin{array}{cccc} k_{1} & k_{2} & p_{1} & p_{2} \end{array}\;\right]=\left[\begin{array}{cccc} -0.1438 & 0.4073 & -0.0003 & -0.0017 \end{array}\right]$$

where ${(C}_{x}{,C}_{y})$ represents the principal point of the distorted image and $f_{x}$ and $f_{y}$ represent the focal lengths in pixels on the x and y directions, with the mean value converted to the actual focal length f in mm via the pixel size:

(15) $$f=s\cdot {(f}_{x}{+f}_{y})\;/\;2=8.3603\;mm$$

**Figure 5:**
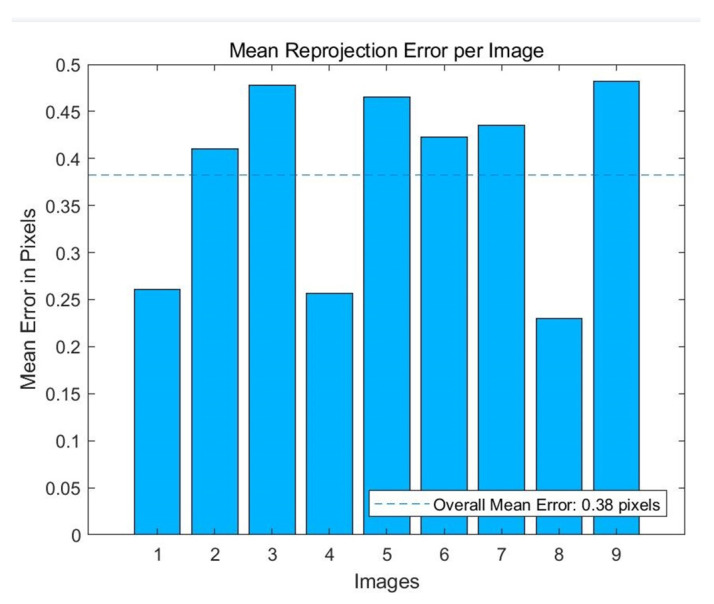
Mean reprojection error of nine images.

$k_{1}$ and $k_{2}$ represent the radial lens distortion parameters and $p_{1}$ and $p_{2}$ represent the tangential lens distortion parameters. Figure 5 indicates that all the mean reprojection errors of the nine images are below 0.5 pixels, and the overall mean error is 0.38 pixels.

According to the above results, image correction is performed using the nonlinear imaging model below:

(16) $$\{\begin{array}{c} x^{\prime }{=x(1+k}_{1}r^{2}{+k}_{2}r^{4}{)+2p}_{1}{y+p}_{2}{(r}^{2}{+2x}^{2})\\ y^{\prime }{=y(1+k}_{1}r^{2}{+k}_{2}r^{4}{)+2p}_{1}{x+p}_{2}{(r}^{2}{+2x}^{2}) \end{array}\;{,\;r}^{2}{=x}^{2}{+y}^{2}$$

where ${(x}^{\prime }{,y}^{\prime })$ and (x,y) are the distorted and undistorted points in the camera coordinate system, which satisfy the following relationship with their corresponding points ${(u}^{\prime }{,v}^{\prime })$ and (u,v) in the image coordinate system:

(17) $$\{\begin{array}{c} x=(u\;–{\;C}_{x}{)\;/\;f}_{x}\\ y=(v\;–{\;C}_{y}{)\;/\;f}_{y} \end{array}$$

(18) $$\{\begin{array}{c} u^{\prime }{=f}_{x}x^{\prime }{+C}_{x}\\ u^{\prime }{=f}_{y}y^{\prime }{+C}_{y} \end{array}$$

The above three equations establish the coordinate transformation relation between distorted and undistorted images.

### 3.3. Calibration of Eccentric Distances

The eccentric distances below are defined as the 3-D distances between the optical centers of the camera (C) and the LDM (L) shown in Figure 3b, which are manually measured with a Vernier caliper according to their marked positions after system integration.

(19) $$\{\begin{array}{c} \Delta X=45\;\mathrm{mm}\\ \Delta Y=35\;\mathrm{mm}\\ \Delta Z=5\;\mathrm{mm} \end{array}$$

The key point that should be taken into account is their effect on the elevation of the camera on the object plane:

(20) $$Z_{C}=d\cdot {\sin\omega }_{L\;}-\Delta Y\cdot {\sin\omega }_{L}{+Z}_{S}$$

where d and $\omega_{L}$ represent the slope distance and pitch angle of LDM relative to the object plane, respectively. In practice, the d should be measured with LDM by aiming at the water boundary rather than the water surface. The $Z_{S}$ is defined as the height of the measured water surface relative to the reference point R, which is supposed to be zero in this case (i.e., point R coincides with point F). In practice, it can be simultaneously measured with the same camera using image-based methods [31].

### 3.4. Calibration of Eccentric Angles

**Figure 6:**
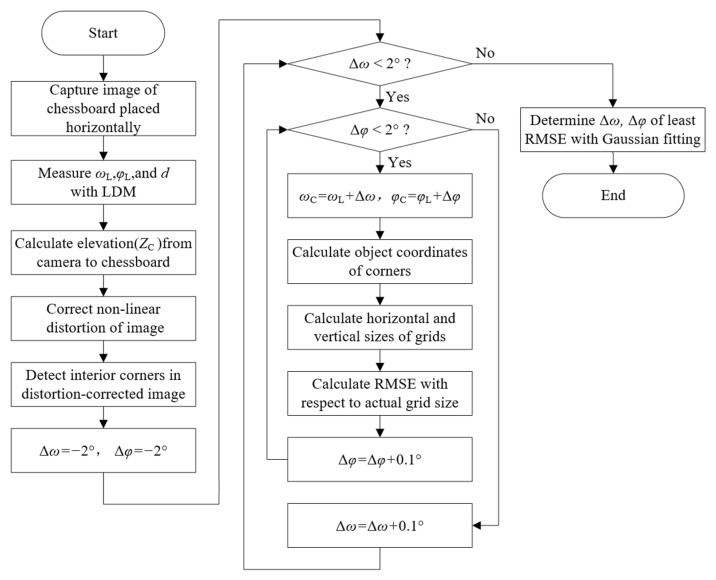
Flow chart of eccentric angles calibration.

The eccentric angles (Δκ, Δω and Δφ) are defined as the differences in three rotation angles between the camera and the LDM, where Δκ→0, Δω, and Δφ are calibrated according to the flow chart shown in Figure 6. The basic principle is to determine the least root-mean-square error (RMSE) of the planar grid size:

(21) $$\left(\Delta \omega \;,\;\Delta \varphi )=\arg\;\min[E(\Delta \omega \;,\;\Delta \varphi )]=\arg\;\min[\sqrt{{(E}_{X}{}^{2}{+E}_{Y}{}^{2})\;/\;2}\right]$$

where $E_{X}$ and $E_{Y}$ represent the total RMSEs of measured grid sizes in X and Y directions, respectively:

(22) $$\{\begin{array}{c} E_{X}=\sqrt{\frac{1}{\left(M\;–\;1\right)N}\overset{M-1}{\underset{i=1}{\sum }}\overset{N}{\underset{j=1}{\sum }}{\left[\left(X_{i+1,j}-X_{I,j}\right)\;–{\;D}_{X}\right]}^{2}}\\ E_{Y}=\sqrt{\frac{1}{M\left(N\;–\;1\right)}\overset{M}{\underset{i=1}{\sum }}\overset{N-1}{\underset{j=1}{\sum }}{\left[\left(Y_{I,j+1}-Y_{I,j}\right)\;–{\;D}_{Y}\right]}^{2}} \end{array}$$

M and N are the number of interior corners on the chessboard, $\left(X_{i,j}{,Y}_{i,j}\right)$ represent their object coordinates, and $D_{X}$, $D_{Y}$ are the actual sizes of the grid. Considering the system integration precision of the device, the minimum value of E(Δω, Δφ) was firstly searched within the ±2° neighborhood of $\omega_{L}$ and $\varphi_{L}$ with a step of 0.1°, then refined with the three-point Gaussian fitting method to make the error of eccentric angles less than 0.1°.

**Figure 7:**
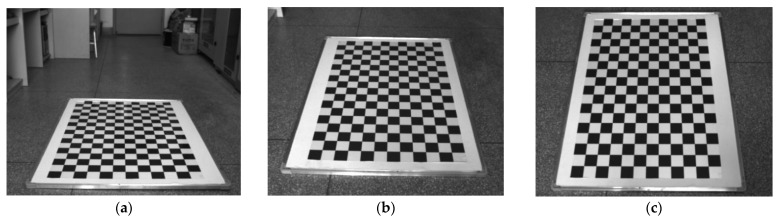
Calibration of eccentric angles under different shooting angles, showing (**a**) set 1, (**b**) set 2, and (**c**) set 3.

To analyze the sensitivity of the calibration method to the pitch angle (ω), three sets of chessboard images were captured for testing (Figure 7). The chessboard was the same as the one used in Figure 4, which was placed horizontally on the ground. The measuring device was installed on a tripod and adjusted to be level according to the bubble. Table 1 indicates that the elevation bias caused by the eccentric distances ΔZ and ΔY was within 4 mm in this case. It is negligible when it is much less than d. Table 2 compares the measurement errors with and without the calibration of eccentric angles. In the set which directly used $\omega_{L}$ and $\varphi_{L}$ for measurements, the RMSE decreased significantly with the increase in pitch angle. This was caused by a reduction in image resolution due to image perspective distortion, especially in the far-field. In view of this, the chessboard should be placed in the near-field of the image when the calibration is performed at a small pitch angle such as in Figure 7a. The RMSEs calculated with eccentric angle calibration decrease to a similar level with a difference of less than 0.05 mm. This is attributed to the global adjustment effect of the least square method. Note that the RMSEs include the errors of corner detection and angle measurement. According to the grid size in images, the mean relative error is approximately 2%, which is similar to the mean reprojection error in Figure 5. Additionally, it was found that the differences in eccentric angles preliminarily calibrated in the three sets were all within 0.1°, which agrees with the searching step. In comparison, the differences decrease to 0.05° after Gaussian fitting, and the measuring precision is further improved for set one with a small pitch angle. Here, the calibration results of set three with the smallest image perspective distortion are chosen for the following measurement, and the corresponding relative error of 2.03% is taken as the uncertainty of the scaling factor u(S).

## 4. Experiment in Flume

### 4.1. Experiment Settings

**Figure 8:**
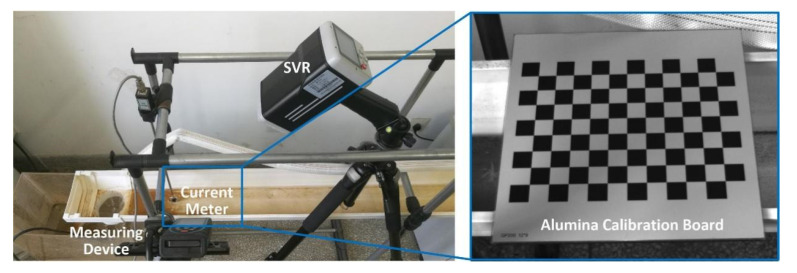
Flume experiment settings.

An experiment was conducted in a recirculating flume to evaluate the proposed method in small-scale surface velocity field measurement. The 4 m-long flume was simply built using PVC rain gutters, as shown in Figure 8. Its section was trapezoid with the upper width of 160 mm, the lower width of 100 mm and the height of 80 mm. The upstream inlet boundary condition was uniform inflow velocity, the downstream outlet boundary condition was free outflow, and the wall boundary condition was non-slip solid wall. The measuring device was installed 0.2 m upstream of the outlet. Since the parameters of the lens cannot be changed after calibration, the height of device was adjusted to make imaging clear. Note that the imaging model in Figure 2 takes the measuring plane as the reference system, while the angle measurement of LDM is based on the geodetic reference system. This difference should be taken into account when calculating the camera orientations. In the experiment, the angle measurements of LDM were $\omega_{L}=59.8^{{}^{\circ}}$ and $\varphi_{L}=-1.2^{{}^{\circ}}$. The vertical and horizontal slopes of the flume were 1.23° and 0°, respectively. It resulted in $\omega_{C}=59.272^{{}^{\circ}}$ and $\varphi_{C}=-1.927^{{}^{\circ}}$ with eccentric angle and slope compensation. The elevation from the camera to the flume bottom is calculated in Table 3. As the water surface was approximately 8 mm from the flume bottom (i.e., Δl=8 mm), the actual camera elevation was 0.541 m according to (20). The size of the effective measurement area shown in Figure 8 was approximately 640 × 480 pixels.

### 4.2. Comparison of In Situ and Non-In Situ Calibration

In order to verify whether the calibration results can be transferred into the actual measuring environment, a high-precision alumina calibration board was used for in situ calibration. There were 12 × 9 grids with a side length of 15 mm and an accuracy of 0.01 mm on the board. As its size was 200 × 200 mm, which was larger than the water surface to be measured, it was set on the top of the flume. Considering the thickness of the calibration board (i.e., Δl=3 mm), the actual elevation of the camera relative to the board was 0.464 m (Table 4). In comparison with the calibration results of set 3 in Table 2, the differences of Δω and Δφ shown in Table 5 were both within the reasonable error range of 0.1°. Therefore, this proves that the calibration method of this integrated measuring device is practically operable. In addition, the Root Mean Square Error (RMSE) relative to the grid size was 0.73%. Note that this value is approximately 1/3 of the relative error given in Section 3.4. It was found that the mean spatial resolutions of the two cases (0.286 × 0.364 mm/pixel vis-à-vis 0.849 × 1.224 mm/pixel) also approximately met this ratio. As a result, it is considered to be a major error source of measurement alongside the shooting angle.

### 4.3. Velocity Measurement

The flow rate remained stable during the experiment. For the convenience of flow field observation with human eyes, a small number of sediments and surfactants were seeded to the water to produce distinguishable foam patterns on the free surface. The camera continuously captured images for 100 s with a frame rate of 100 fps. The total 10,000 frames were evenly divided into 40 groups for analysis.

**Figure 9:**
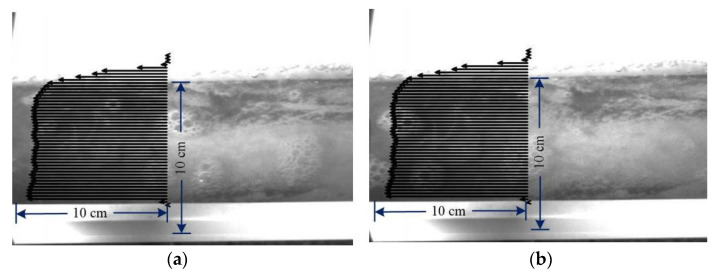
Visualization of optical flow field with different background image, showing (**a**) 18th frame and (**b**) 28th frame.

**Figure 10:**
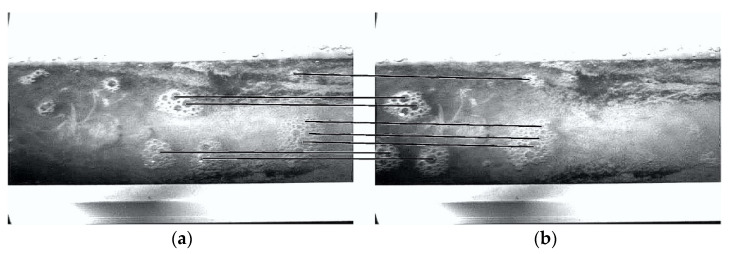
Matching point pairs manually labeled in 18th frame (**a**) and 28th frame (**b**).

Firstly, motion estimation with FFT-STIV was tested. A total of 50 testing lines with a length of 255 pixels and a space interval of 50 pixels were set across the section of flume in each frame. Figure 9 visualizes the time-averaged optical flow field obtained by group 1, where the black arrow line represents the optical flow velocity $v_{x}$ (pixel/frame) corresponding to each testing line. For easy observation, the arrow lines were enlarged by 10 times, and two frames (18^th^ and 28^th^) with uniform and clear foam patterns were selected as background images. The central coordinates $\left(x_{1}{,\;y}_{1}\right)$ and $\left(x_{2}{,\;y}_{2}\right)$ of eight foams in the two frames were manually labeled as the corresponding points (Figure 10). Table 6 provides the displacements $\left(d_{x}{,\;d}_{y}\right)$ of the corresponding points and compares their optical flow velocities in the x direction (denoted as $u_{x}$) with the FFT-STIV measurements. It shows that there is a good consistency between them. Here, the maximum relative error of 3.24% is taken as the uncertainty of motion estimation u(v), which includes errors in manual labeling.

**Figure 11:**
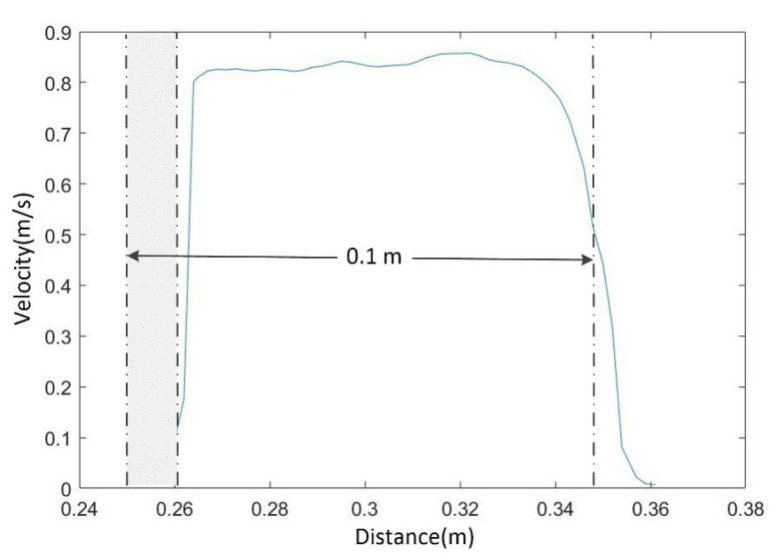
Cross-section velocity distribution averaged with a total of 40 calibrated velocity fields.

Secondly, velocity calibration with DSO was tested. Figure 11 presents the cross-section velocity distribution calculated with a total of 40 groups of calibrated velocity fields. It agreed with the pattern of velocity distribution in trapezoidal open channels [32]; the velocities varied dramatically near two side walls due to the frictional effect, but remained stable after a certain distance from them. In theory, the average velocity at the midstream (0.3 m) should be maximum; in this case, the measured velocity was approximately 0.02 m/s lower than those near 0.32 m. This is because the large foams were mainly concentrated in the midstream, which did not follow the surface flow as well as the small ones did. The starting distances of each testing line measured with the DSO method ranged from 0.260 m to 0.361 m. The field of view within 0.26 m was occluded by the right-side wall due to the limited observation angle, resulting an unmeasurable area of 0.012 m (shaded area in Figure 11). The measured distance between the two lower boundaries of the trapezoid section was 0.1 m, which was consistent with the true width of the bottom surface of the flume. The conclusion above verifies that the DSO method is effective for free-surface measurement and velocity calibration.

### 4.4. Uncertainty Evaluation

The flow rate remained stable during the experiment. For the convenience of flow field observation with human eyes, the uncertainty evaluation of real flow field measurement has always been a difficulty in PIV related studies. In this experiment, a propeller current meter (PCM) and a surface velocity radar (SVR) were set up to carry out a comparison measurement. The PCM was fixed at the midstream (0.3 m) to measure its velocity on the water surface. The average velocity measured over a period of 100 s was 0.496 m/s. The SVR was set on one side of the flume, approximately 0.65 m above the water surface. Its pitch angle was 52° and the beam heading angle relative to the flow direction was 5°. The average velocity measured over a period of 100 s was 0.44 m/s, with a maximum of 0.63 m/s and a minimum of 0.38 m/s. Obviously, the measurements of both instruments were much lower than those of the DSO-based FFT-STIV method. The reasons for this are: (a) the water depth was too shallow for the propeller to completely submerge under the water, which results in low spin number; and (b), the water was clear and shallow enough for the electromagnetic wave to pass through the free surface, enough to reach the bottom and reflect back. The actual measured velocity amounts to the depth-averaged one, rather than the superficial one. Therefore, both instruments have limitations under the experimental conditions and cannot be used as effective references for an uncertainty evaluation.

In view of this, the repeatability precision of the DSO-based FFT-STIV method was assessed by analyzing the 40 groups of velocity measurements in this study, as shown in Table 7. For the testing lines in the bottom inner zone (0.265~0.341 m), the deviation between their mean and median values was within 0.004 m/s. The standard deviation (SD) was less than 0.03 m/s, and the relative standard deviation (RSD) was less than 5%, among which 85% were less than 2%. For each testing line, its 40 measurements were firstly sorted into ascending order according to the absolute value of the RSD. The 38th value was then taken as the relative error with a cumulative frequency of 95%, which ranges from 1.66% to 7.53%. Considering factors such as velocity pulsation, this repeatability precision is reasonable for free-surface velocity measurement. In addition, the cumulative frequency errors (CFEs) are approximately twice the relative standard deviations. It can be assumed that the measurement error obeys the normal distribution law. In contrast, the random coarse error caused by strong flow turbulence and poor tracer conditions in the bottom outer zone can be beyond ±10%. To indicate the relationship between spatial resolution and measurement error, Table 7 also gives the scaling factors of each testing line. Although the spatial resolution increases with the distance of the testing line from the camera, the measurement error does not show an increasing trend. This is because of the global adjustment effect of the least square method which is discussed in Section 3.4. Moreover, $S_{x}$ changes less significantly than $S_{v}$, indicating that the x direction of the image is less affected by perspective distortion. It can be inferred that the accuracy of velocity calibration is higher than that of the starting distance when the optical axis of the camera is perpendicular to the direction of water flow.

According to (1), the physical velocity V only depends on the optical flow velocity v and scaling factor S. Considering the independence of their errors, here we bring the previously mentioned u(S) and u(v) into the following equation to estimate the combined standard uncertainty of velocity:

(23) $$\;u\;\left(V\right)=\sqrt{u^{2}\left(v\right){+u}^{2}\left(S\right)\;}=\sqrt{{\left(3.24\%\right)}^{2}+{\left(2.03\%\right)}^{2}\;}=3.823\%$$

It can be found that this value is close to the measured maximum RSD of 3.81% (testing line 41) in the bottom inner zone. According to the normal distribution law of measurement error, the extended uncertainty of 95% confidence level is estimated by using two times the combined standard uncertainty:

(24) $$U_{95}\left(V\right)=2\cdot u\left(V\right)=7.646\%$$

This value is also close to the CFE (7.53%) of testing line 41. This observation implies that the uncertainty synthesis method is feasible for the evaluation of DSO-based FFT-STIV.

## 5. Conclusions

The DSO-based STIV is proposed to realize 1-D free-surface velocity measurement under an oblique shooting angle. It also provides a GCPs-free solution to the problem of velocity calibration in planar PIV applications where reference objects cannot be deployed in situ. On the basis of the traditional collinear equation, lens distortion, oblique shooting angle, water level variation, and water surface slope are introduced to build an accurate and reasonably simplified imaging model to describe the coordinate transformation relation between image and object planes. The LDM-supported measuring device is low-cost and easy to construct. It only needs to be calibrated once if the camera’s intrinsic parameters and relative position within the LDM remain constant. The comparison of non-in situ and in situ calibration results verify good transferability of the calibration method. The effectiveness of motion estimation with FFT-STIV and velocity calibration with DSO were verified step by step based on the designed flume experiment. Finally, the combined uncertainty of velocity measurement was evaluated, which provided an effective means for error analysis when comparing measurement failures. Overall, the DSO-STIV proved superior to propeller current meters and surface velocity radars in surface velocity measurements in extremely shallow water conditions such as those in this study. It not only has high spatial resolution up to a single pixel, but also has good measuring precision with the standard uncertainty controlled within 5% in small-scale applications. In future work, the DSO-STIV will be applied to natural rivers to evaluate the sensitivity and uncertainty of large-scale river surfaces.

## Figures and Tables

**Table 1:** Calibration results of eccentric distances and camera elevation.

Set	*d* (m)	$\omega_{L}\;({}^{\circ})$	ΔZ (m)	ΔY (m)	$Z_{C}\;(m)$
1	2.414	19.6	0.005	0.025	0.806
2	1.778	41.3	0.005	0.025	1.161
3	1.784	59.1	0.005	0.025	1.512

**Table 2:** Calibration results of eccentric angles and camera orientations.

Set	Without Calibration			Without Gaussian Fitting			With Gaussian Fitting				
	$\omega_{L}$ (°)	$\varphi_{L}$ (°)	*E* (mm)	$\omega_{C}\;({}^{\circ})$	$\varphi_{C}({}^{\circ})$	*E* (mm)	$\omega_{C}\;({}^{\circ})$	$\varphi_{C}\;({}^{\circ})$	*E* (mm)	Δω (°)	Δφ (°)
1	19.6	0.6	2.298	19.1	1.1	1.249	19.070	1.064	1.244	−0.530	0.464
2	41.3	0.1	1.716	40.7	0.5	1.205	40.716	0.523	1.205	−0.584	0.423
3	59.1	−0.6	1.357	58.6	−0.1	1.217	58.572	−0.097	1.217	−0.528	0.503

**Table 3:** Calibration results of eccentric distances and camera elevation to flume bottom.

Item	d (m)	$\omega_{L}$ (°)	$Z_{L}\;(m)$	ΔZ (m)	ΔY (m)	$Z_{C}\;(m)$
**Value**	0.657	59.8	0.568	0.005	0.025	0.549

**Table 4:** Calibration results of eccentric distances and camera elevation to the calibration board.

Item	d (m)	$\omega_{L}\;({}^{\circ})$	ΔZ (m)	ΔY (m)	$Z_{C}\;(m)$
**Value**	0.560	59.8	0.005	0.025	0.464

**Table 5:** Calibration results of eccentric angles and camera orientation to the calibration board.

	Without Calibration			Without Gaussian Fitting			With Gaussian Fitting				
Item	$\omega_{L}$ (°)	$\varphi_{L}$ (°)	*E* (mm)	$\omega_{C}\;({}^{\circ})$	$\varphi_{C}\;({}^{\circ})$	*E* (mm)	$\omega_{C}\;({}^{\circ})$	$\varphi_{C}\;({}^{\circ})$	*E* (mm)	Δω (°)	Δφ (°)
**Value**	59.8	−2.4	0.175	59.3	−2.0	0.110	59.2 72	−1.958	0.109	−0.528	0.442

**Table 6:** Comparison of visual measurements and FFT-STIV measurements.

Points	1	2	3	4	5	6	7	8
(*x*_1_, *y*_1_) (pixel)	(466, 147)	(272, 184)	(291, 195)	(486, 224)	(488, 243)	(480, 258)	(249, 272)	(320, 284)
(*x*_2_, *y*_2_) (pixel)	(251, 156)	(48, 187)	(64, 199)	(269, 233)	(269, 251)	(257, 263)	(22, 278)	(90, 285)
(*d_x_*, *d_y_*) (pixel)	(−215, 9)	(−224, 3)	(−225, 4)	(−217, 9)	(−219, 8)	(−223, 5)	(−227, 6)	(−230, 1)
*u_x_* (piels/frame)	21.5	22.4	22.5	21.7	21.9	22.3	22.7	23.0
*v_x_* (pixels/frame)	21.770	22.192	21.770	21.924	22.051	22.192	22.542	22.279
*E*_r_ (%)	1.26%	−0.93%	−3.24%	1.03%	0.69%	−0.48%	−0.70%	−3.13%

**Table 7:** Statistical results of velocity measurements.

No	Starting Distance (m)	Scaling Factor		Velocity						
		*S_x_* (mm/pixel)	*S_y_* (mm/pixel)	Mean (m/s)	Median (m/s)	SD (m/s)	RSD (%)	Maximum RSD (%)	Minimum RSD (%)	CFE (%)
1	0.26	0.346	0.381	0.094	0.007	0.099	105.79	158.27	−94.66	143.33
2	0.262	0.347	0.382	0.789	0.796	0.079	9.96	16.77	−56.51	102.75
3	0.264	0.347	0.384	0.803	0.798	0.029	3.64	10.83	−7.60	6.74
4	0.265	0.348	0.385	0.817	0.813	0.025	3.01	7.04	−9.23	6.03
5	0.267	0.348	0.386	0.827	0.829	0.021	2.52	5.36	−7.58	3.62
6	0.269	0.349	0.387	0.822	0.819	0.016	1.98	5.33	−4.28	3.00
7	0.271	0.35	0.389	0.826	0.823	0.016	1.95	5.75	−4.29	2.56
8	0.273	0.35	0.39	0.827	0.826	0.015	1.84	4.01	−3.85	3.26
9	0.275	0.351	0.391	0.819	0.818	0.017	2.11	3.85	−4.08	3.25
10	0.277	0.351	0.392	0.824	0.824	0.014	1.64	4.36	−3.90	3.13
11	0.279	0.352	0.394	0.825	0.824	0.012	1.49	3.43	−3.00	2.89
12	0.281	0.352	0.395	0.826	0.824	0.011	1.31	3.52	−2.53	2.29
13	0.283	0.353	0.396	0.823	0.824	0.012	1.48	3.90	−3.27	2.23
14	0.285	0.354	0.398	0.821	0.819	0.013	1.61	3.92	−3.63	2.60
15	0.287	0.354	0.399	0.827	0.825	0.011	1.28	3.04	−2.16	2.60
16	0.289	0.355	0.4	0.831	0.829	0.011	1.30	4.12	−1.42	1.98
17	0.291	0.355	0.402	0.832	0.832	0.012	1.46	3.99	−3.82	2.52
18	0.293	0.356	0.403	0.841	0.842	0.011	1.28	2.36	−1.92	2.35
19	0.295	0.357	0.404	0.842	0.841	0.011	1.33	4.13	−1.81	2.22
20	0.297	0.357	0.406	0.836	0.835	0.010	1.23	3.05	−3.66	1.87
21	0.299	0.358	0.407	0.833	0.832	0.012	1.42	3.80	−2.80	1.86
22	0.301	0.358	0.408	0.829	0.828	0.011	1.34	3.26	−2.77	2.31
23	0.303	0.359	0.41	0.832	0.834	0.014	1.63	4.16	−3.05	2.27
24	0.305	0.36	0.411	0.833	0.833	0.010	1.24	3.65	−2.47	2.40
25	0.307	0.36	0.413	0.833	0.833	0.011	1.28	4.17	−3.51	2.57
26	0.309	0.361	0.414	0.835	0.834	0.009	1.09	4.04	−2.30	1.66
27	0.311	0.361	0.415	0.845	0.844	0.011	1.29	3.20	−2.36	1.99
28	0.313	0.362	0.417	0.852	0.851	0.012	1.41	2.56	−2.84	2.00
29	0.316	0.363	0.418	0.859	0.856	0.010	1.12	2.46	−2.08	2.03
30	0.318	0.363	0.42	0.854	0.855	0.010	1.14	2.72	−2.08	1.99
31	0.32	0.364	0.421	0.859	0.858	0.011	1.28	3.01	−2.11	1.78
32	0.322	0.365	0.423	0.856	0.855	0.009	1.08	2.89	−1.78	1.71
33	0.324	0.365	0.424	0.847	0.848	0.011	1.28	2.80	−2.75	1.70
34	0.326	0.366	0.425	0.842	0.844	0.011	1.28	2.73	−3.21	2.05
35	0.328	0.366	0.427	0.840	0.840	0.012	1.44	2.41	−4.14	2.33
36	0.33	0.367	0.428	0.837	0.841	0.015	1.76	3.18	−5.65	2.23
37	0.333	0.368	0.43	0.827	0.825	0.014	1.74	4.38	−4.57	2.70
38	0.335	0.368	0.431	0.815	0.817	0.014	1.68	3.39	−5.07	2.56
39	0.337	0.369	0.433	0.799	0.797	0.019	2.32	4.08	−5.30	3.86
40	0.339	0.37	0.434	0.778	0.776	0.024	3.11	6.91	−7.23	5.07
41	0.341	0.37	0.436	0.755	0.759	0.029	3.81	5.99	−9.51	7.53
42	0.343	0.371	0.437	0.702	0.714	0.040	5.76	8.97	−10.83	7.84
43	0.346	0.372	0.439	0.581	0.578	0.057	9.80	19.50	−23.20	13.94
44	0.348	0.372	0.44	0.462	0.462	0.033	7.17	14.38	−10.96	12.26
45	0.35	0.373	0.442	0.433	0.424	0.052	12.05	57.81	−15.43	11.26
46	0.352	0.374	0.444	0.293	0.381	0.162	55.43	65.77	−97.61	86.80
47	0.354	0.374	0.445	0.049	0.008	0.060	121.60	272.90	−85.74	163.30
48	0.357	0.375	0.447	0.016	0.007	0.031	188.18	843.68	−57.38	106.42
49	0.359	0.376	0.448	0.007	0.007	0	0	0	0	0
50	0.361	0.376	0.45	0.007	0.007	0	0	0	0	0

## Data Availability

All the data presented in this study are available in this article.
